# Inhomogeneity of epidemic spreading

**DOI:** 10.1063/1.3445630

**Published:** 2010-06-23

**Authors:** Zhenzhen Liu, Xingyuan Wang, Mogei Wang

**Affiliations:** School of Electronic and Information Engineering, Dalian University of Technology, Liaoning 116024, People’s Republic of China

## Abstract

In this study, we use the characteristic infected cluster size to investigate the inhomogeneity of the epidemic spreading in static and dynamic complex networks. The simulation results show that the epidemic spreads inhomogeneously in both cases. Also, the inhomogeneity of the epidemic spreading becomes smaller with increasing speed of moving individuals and almost disappears when the speed is high enough.

The epidemic has always been a threat to the human health. The rule of the epidemic spreading as an important issue has been quite extensively studied for many years, but has not been mastered well. The epidemic spreads by the interaction between people, and complex networks, whose nodes represent individuals or organizations and link the interactions among them, can characterize this interpersonal interaction perfectly. In order to conveniently study the epidemic spreading, researchers usually adopt the homogeneous network structure and the homogeneous mixing hypotheses. Recently, researchers began to discuss the credibility of the homogeneous network structure hypothesis and consider the inhomogeneity of the network structure. However, the credibility of the homogeneous mixing hypothesis has not been well discussed until now. In this paper, we try to discuss this credibility and carry out numerical simulations on an epidemic spreading model. The results show that the epidemic spreading is inhomogeneous whether the individuals are stationary or moving. However, the inhomogeneity decreases with the increase of individuals’ speed, and almost disappears when the speed is high enough.

## I. INTRODUCTION

In recent years, physicists and biologists have been attracted to the spreading of the epidemic. Undoubtedly, the successive large-scale outbreaks of SARS (Severe Acute Respiratory Syndrome) and H1N1 make them more enthusiastic about this study. Complex networks as a new branch of statistical physics provide a reliable model for the intensive study of the epidemic spreading. When studying the epidemic spreading in complex networks, researchers usually adopt the homogeneous network structure^1–8^ and homogeneous mixing^3,9–11^ hypotheses. As research progressed, researchers began to discuss the credibility of the homogeneous network structure hypothesis and take into account the inhomogeneity of the network structure.^1–5,12^ In this study, we try to check the credibility of the homogeneous mixing hypothesis, meaning that each infected individual has the same probability of contacting with any susceptible (healthy) individual,^13^ which has not been well discussed until now.

We will focus on the susceptible-infected-susceptible (SIS) model^14^ on complex networks. Here each node in the network represents an individual, and each link represents a connection along which the epidemic can spread. A susceptible individual can be infected by its infected neighbors with some probability, and the infected individual can become susceptible with other probability. As the infected individuals always infect their neighbors, we predict that the spreading of the epidemic may actually be inhomogeneous. That is, the probabilities of infected individuals connecting with susceptible ones differ significantly. To investigate the inhomogeneity, we perform large scale numerical simulations on static and dynamic networks. In these simulations, we study two spreading modes of the SIS model. In one mode, the probability that a susceptible individual is infected is unrelated to the number of its infected neighbors. In another mode, the probability increases with the number.

In this work, the inhomogeneity of the epidemic spreading is characterized by the characteristic infected cluster size (hereinafter referred to as “CICS”), where the cluster is the subnet whose nodes are connected^13,15^ (i.e., from any node, one can reach any other node along links in the subnet), the infected cluster only includes infected individuals, and the CICS is the typical size of the largest infected cluster, namely, the number of infected individuals of the largest infected cluster. The simulation results show that the infected individuals are always distributed inhomogeneously and prone to gather into large clusters, even if they walk randomly. More interestingly, the inhomogeneity of the epidemic spreading decreases with increasing speed of the individuals, and the epidemic nearly spreads homogeneously when the moving speed is high enough.

## II. MODEL

In our model, N individuals walk randomly in a square of linear size L with periodic boundary conditions, and they are distributed randomly in the square initially. The $x_{i}\left(t\right)$ and $\theta_{i}\left(t\right)$ are the position and motion direction of the ith individual at time t. Then, at time t+Δt, they are updated according to

{xi(t+Δt)=xi(t)+vi(t)Δtθi(t+Δt)=ξi}, (1)

where $v_{i}\left(t\right)=\left(v\;\text{cos}\;\theta_{i}\left(t\right),v\;\text{sin}\;\theta_{i}\left(t\right)\right)$ is the velocity of the ith individual at time t, v is the speed which is the modulus of the velocity. The speed v is same for all individuals and remains constant in motion. $\xi_{i}$ follows the uniform distribution in [−π,π]. Δt is the update interval and is set to 1. If $\left|x_{i}\left(t\right)-x_{j}\left(t\right)\right|\leq r_{0},i\neq j$, the jth (ith) individual is called a neighbor of the ith (jth) at time t, where $r_{0}$ is the interaction radius. This means that there is a link between the ith and jth nodes in the corresponding network.

We assume that the update of the individuals’ positions and states is simultaneous with the fixed time interval Δt. Each individual has two states: susceptible (S) and infected (I). The next state of each individual depends on its current state and its neighbors’ states. Concretely, if the ith individual is infected currently, then it is cured and becomes susceptible at the next time step with probability β; if it is susceptible currently, then it can be infected by its infected neighbors in either of two modes. In mode 1, the ith individual is infected with probability α if it has k(i)>0 infected neighbors. In mode 2, it is infected with probability $1-{\left(1-\alpha \right)}^{k\left(i\right)}$. Apparently, each infected neighbor of the ith individual infects it independently.

Researchers have analyzed the epidemic thresholds of mode 1 in various complex networks^1–3,16^ and have shown that the degree distribution of the network has a significant impact on the epidemic spreading. Liu *et al.*^17^ investigated the epidemic spreading of mode 2 in community networks. The epidemic spreading of mode 2 in dynamic networks has been explored by Frasca *et al.*^18^ recently. In this work, the mode 1 and mode 2 are both studied. Without lack of generality, we set β=1, as it only affects the time scale of the infection evolution.^19^ Considering the reality, we will not set α to a large value. As a result, the infected individuals can gather into several clusters rather than one. Then, the homogeneity of the epidemic spreading can be explored by analyzing and comparing the CICSs of the different cases.

In the following, the number of individuals N is fixed to 100. $\rho =N/L^{2}$ is denoted as the density of the network. The size of the square L is measured in units of the interaction radius $r_{0}$ $\left(r_{0}=1\right)$. As we can see, L is critical for determining the network structure. If L approaches to $r_{0}=1$, most individuals are connected together. Then, too many individuals will be infected persistently. By contrast, if L is much larger than $r_{0}=1$, individuals have few opportunities to interact with each other. The network is divided into many small clusters, then not many individuals will be infected. As a result, the epidemic will disappear soon.

**FIG. 1.**
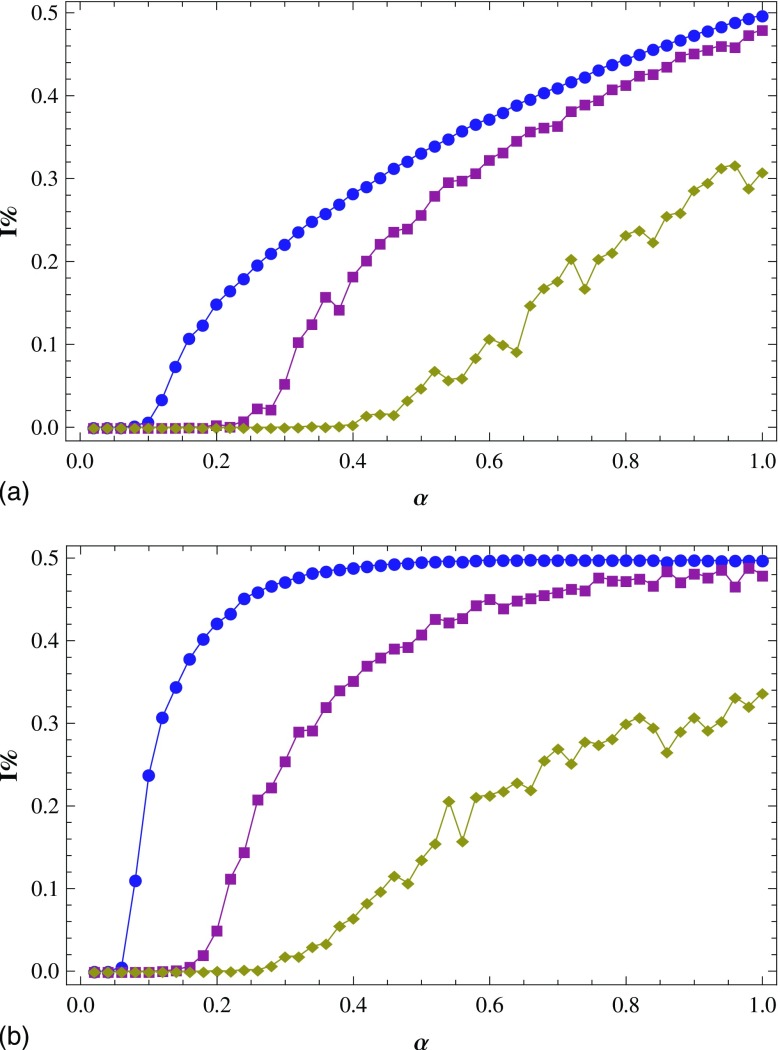
The percentage of infected individuals as a function of α with L=4 (circles), L=7 (squares), and L=10 (diamonds) in mode 1 (a) and mode 2 (b). Results are averaged over 15 runs.

In this work, we only discuss the cases when L is proper. When v=0 (static networks), the percentage of the infected individuals is plotted as a function of α with different L in Fig. 1. As shown in Fig. 1(a) [Fig. 1(b)], when L=10 in mode 1 (mode 2), only when α is above 0.6 (0.45), the epidemic can spread in the network. When L=4 in mode 1 (mode 2), the epidemic persists as long as α is above 0.15 (0.07); about one third (half) of the individuals are infected when α equals to 0.5. Thus, we choose L=7 to ensure that neither the epidemic will disappear, nor too many individuals will be infected, with a large range of α value. Moreover, Fig. 6(b) in Sec. IV indicates that the moving of individuals cannot affect the percentage of the infected individuals in mode 2 and has little influence on the percentage in mode 1. So, we also choose L=7 when v>0 (dynamic networks).

In static networks, the individuals are distributed randomly and keep still over time, so each individual connects to another with the same probability. Thus the degree distribution P(k), as the probability that a node is linked to k other nodes, of static networks is Poissonian. In dynamic networks, the individuals are randomly distributed initially and walk randomly in the square. Therefore, the probability that an individual connects to another is a constant, which equals to the probability of the static case. Then the degree distribution P(k) of dynamic networks is also Poissonian. In Sec. III, we investigate the epidemic spreading in static networks (v=0). Section IV is devoted to the study of the epidemic spreading in dynamic networks (v>0).

## III. EPIDEMIC SPREADING IN STATIC NETWORKS

**FIG. 2.**
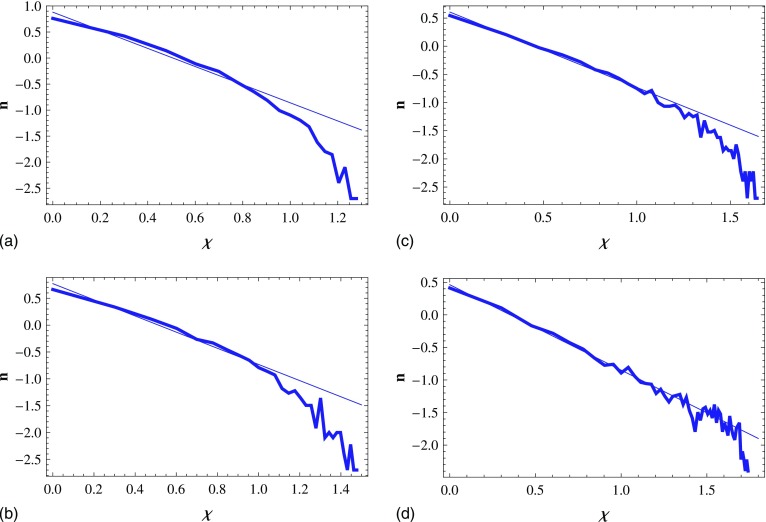
Log-log plot of the infected cluster number n as a function of the infected cluster size χ in a square of size L=7 in homogeneous mode. For N=30 (a), 40 (b), 50 (c), and 60 (d), the infected cluster number decays as a power law for $\chi <\chi_{c}$ and rapidly for $\chi >\chi_{c}$. Results are averaged over 500 runs.

In order to characterize the homogeneity of the epidemic spreading, we introduce the “homogeneous mode.” In this mode, the infected individuals, whose number is $N_{H}$, are randomly scattered in a square of linear size L (L=7). Then their distribution is homogeneous. When the infection density $\rho_{H}=N_{H}/L^{2}$ is not quite large, the distribution of the infected cluster size χ in homogeneous mode is similar to the distribution of the cluster size of the site percolation.^20^ That is, there exists a $\chi_{c}$. When $\chi <\chi_{c}$, the number of infected clusters n decays approximately as a power law in χ, while decaying much faster than a power law for $\chi >\chi_{c}$ (see Fig. 2). In reality, the infected clusters whose sizes are larger than $\chi_{c}$ barely exist, so the infected cluster with the critical infected cluster size $\chi_{c}$ is regarded as the largest infected cluster that can be observed, and $\chi_{c}$ is denoted as the CICS.

With the increase of $\rho_{H}$, a critical infection density $\rho_{c}$ emerges, above which it is possible that all the individuals gather into one cluster. Our experiments show that $\rho_{c}$ is about 1.4. As shown in Fig. 1, the maximum number of infected individuals is about 50, that is, the maximum infection density is about 1 $\left(50/7^{2}\right)$. In such cases, $N_{H}$ infected individuals always gather into more than one cluster. Then we can characterize the homogeneity of their distribution with the CICS. For the convenience of discussion, we denote the CICS of homogeneous mode as $\chi_{H}$.

Correspondingly, the number and density of the infected individuals and the CICS in mode 1 (mode 2) are denoted by $N_{1}$, $\rho_{1}=N_{1}/L^{2}$, and $\chi_{1}$ $\left(N_{2},\rho_{2}=N_{2}/L^{2},\chi_{2}\right)$, respectively. We will see later that when $\rho_{1}=\rho_{2}=\rho_{H}$, $\chi_{1}$, $\chi_{2}$, and $\chi_{H}$ differ significantly, that is, the epidemic spreads inhomogeneously in mode 1 and mode 2. At t=0, a given number of individuals are taken as the seeds of the infection (the proportion is 10%), while all the others start from the susceptible state. Also, all individuals are scattered randomly in the square, namely, the infected and susceptible individuals mix well. After an initial transient process, the systems stabilize in a steady state with a constant average infection density.^1^

**FIG. 3.**
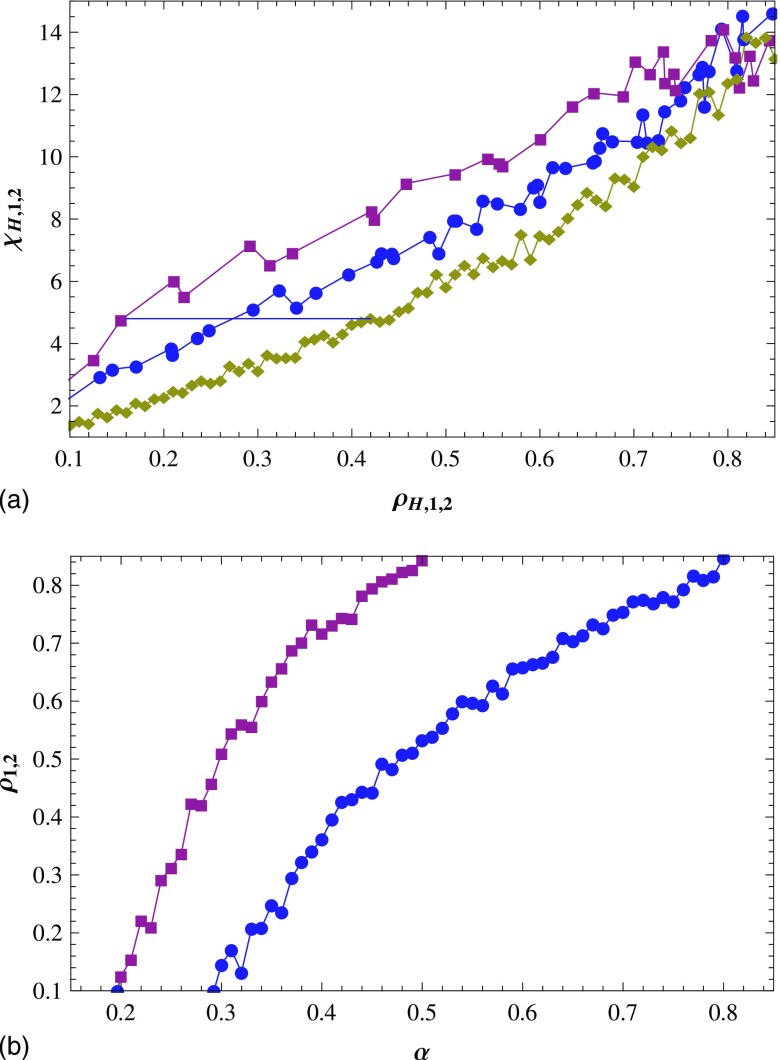
(a) With the change of α, $\chi_{i}$ vs $\rho_{i}$, i=1,2: mode 1 (circles) and mode 2 (squares); $\chi_{H}$ vs $\rho_{H}$ (diamonds) for homogeneous mode. The dashed line is for $\chi_{H}=\chi_{1}=\chi_{2}$. (b) $\rho_{i}$ vs α, i=1,2: mode 1 (circles) and mode 2 (squares). Results are averaged over 15 runs.

For each value of α, we can get the values of $\left(\rho_{1},\chi_{1}\right)$. With changing α, $\chi_{1}$ is plotted as a function of $\rho_{1}$ [the middle curve in Fig. 3(a)]. In the same way, $\chi_{2}$ is plotted as a function of $\rho_{2}$ [the top curve in Fig. 3(a)]. In homogeneous mode, each $\rho_{H}$ (or each $N_{H}=\rho_{H}L^{2}$) corresponds to one $\chi_{H}$, then we can plot $\chi_{H}$ as a function of $\rho_{H}$ [the bottom curve in Fig. 3(a)]. For the sake of getting α value corresponding to a given $\rho_{1}$ $\left(\rho_{2}\right)$, we plot $\rho_{i}$ versus α, i=1,2 in Fig. 3(b).

The initial infection density is 10%, so the epidemic will be persistent with time, only when the infection density is larger than 0.1 after a long spreading. Therefore, we make the epidemic spread in the square for a long time and then discuss the situation when $\rho_{H,1,2}\geq 0.1$ below. As is shown in Fig. 3(a), we draw the following conclusions by contrasting $\chi_{i}$, i=0,1,2 with $\rho_{H}=\rho_{1}=\rho_{2}$. When $\rho_{H,1,2}∊\left[0.1,0.7\right]$, $\chi_{1}$ and $\chi_{2}$ are larger than $\chi_{H}$ significantly, which means that the inhomogeneity exists. Concretely, when $\rho_{H,1,2}$ is near 0.2, $\chi_{1}$ and $\chi_{2}$ are up to 2–3 times $\chi_{H}$, which is a significant difference. With increasing $\rho_{H,1,2}$, the multiple becomes less although the difference between $\chi_{H}$ and $\chi_{1}$ $\left(\chi_{2}\right)$ has little change. Then we can say that the smaller the infection density $\rho_{H,1,2}$, the larger the inhomogeneity of the epidemic spreading.

In these cases, when $\chi_{H}=\chi_{1}=\chi_{2}$, $\rho_{H}$ can reach two or more times $\chi_{1}$ and $\chi_{2}$ [see the line of Fig. 3(a)], that is, the number of infected individuals of homogeneous mode is two or more times that of mode 1 and mode 2. It illustrates that, from another point of view, the epidemic spreading in mode 1 and mode 2 is inhomogeneous. When $\rho_{H,1,2}$ is about 0.8, $\chi_{H}$ and $\chi_{1}$ $\left(\chi_{2}\right)$ are similar, that is, infected individuals are distributed homogeneously in mode 1 and mode 2. However, the α values of mode 1 and mode 2 reach 0.5 and 0.8, respectively [see Fig. 3(b)]. They are so large that we do not discuss this case. In fact, the epidemic always spreads near the infected individuals, that is, the infected individuals only infect their susceptible neighbors, and thus it is a natural thing for epidemic to spread inhomogeneously when the infection density is not so large.

Figure 3(a) also shows that when $\rho_{1}=\rho_{2}$ and $\rho_{H,1,2}∊\left[0.1,0.7\right]$, $\chi_{2}>\chi_{1}$. It means that the epidemic spreading in mode 2 is more inhomogeneous than in mode 1, namely, the infected individuals are much easier to gather into large clusters in mode 2 than in mode 1. In addition, when the values of α are the same, $\rho_{2}>\rho_{1}$, as can be seen from Fig. 3(b). It also demonstrates the same conclusion. The reason is that the probability that a susceptible individual is infected increases with the number of its infected neighbors in mode 2, i.e., the susceptible individuals are more easily infected in mode 2 than in mode 1.

## IV. EPIDEMIC SPREADING IN DYNAMIC NETWORKS

**FIG. 4.**
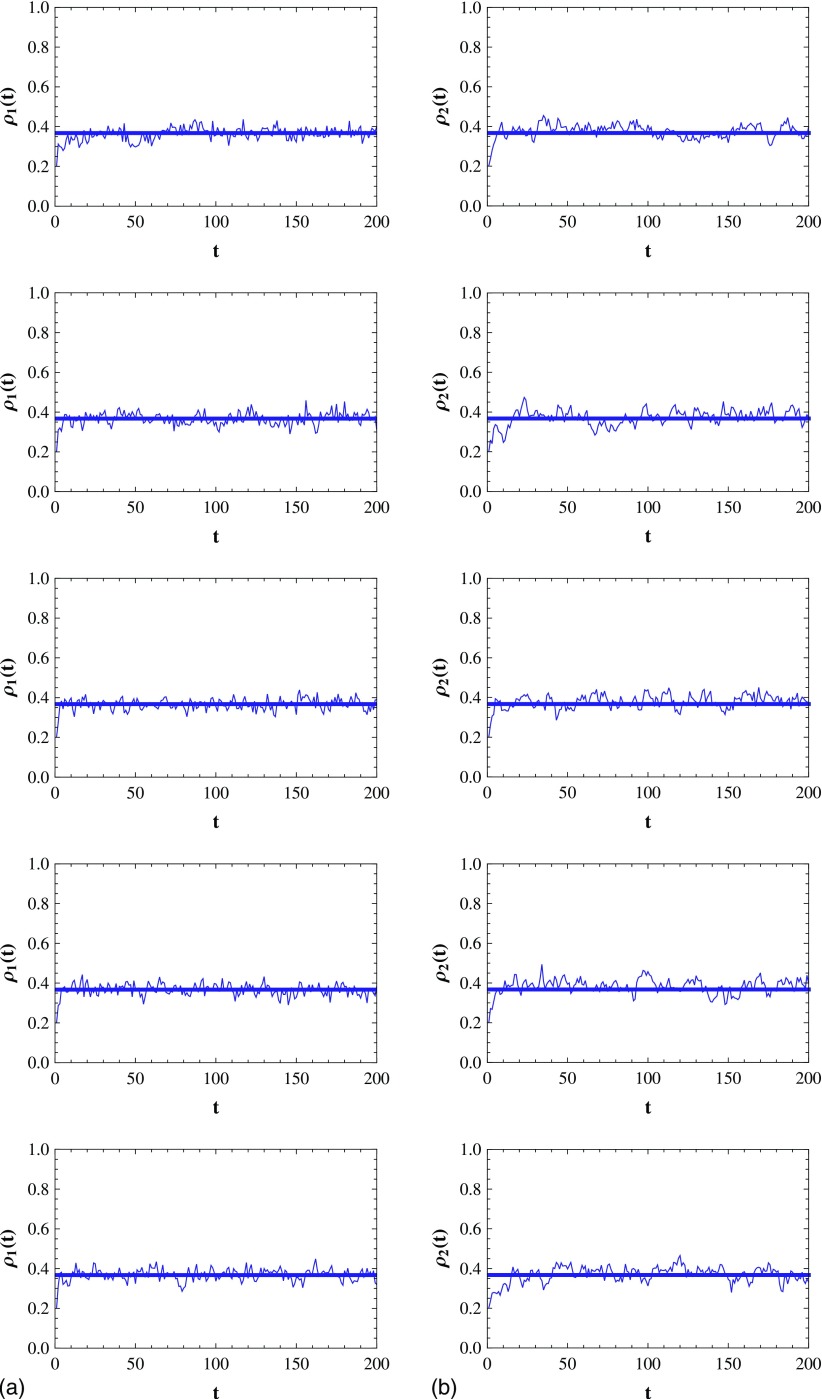
(a) The evolution of the infection density $\rho_{1}$ in mode 1. v=0.2,1,2,6,10 (from top to bottom). Each straight line corresponds to the average infection density after the initial transient process. (b) Same with (a) but for mode 2. Results are averaged over 15 runs.

**FIG. 5.**
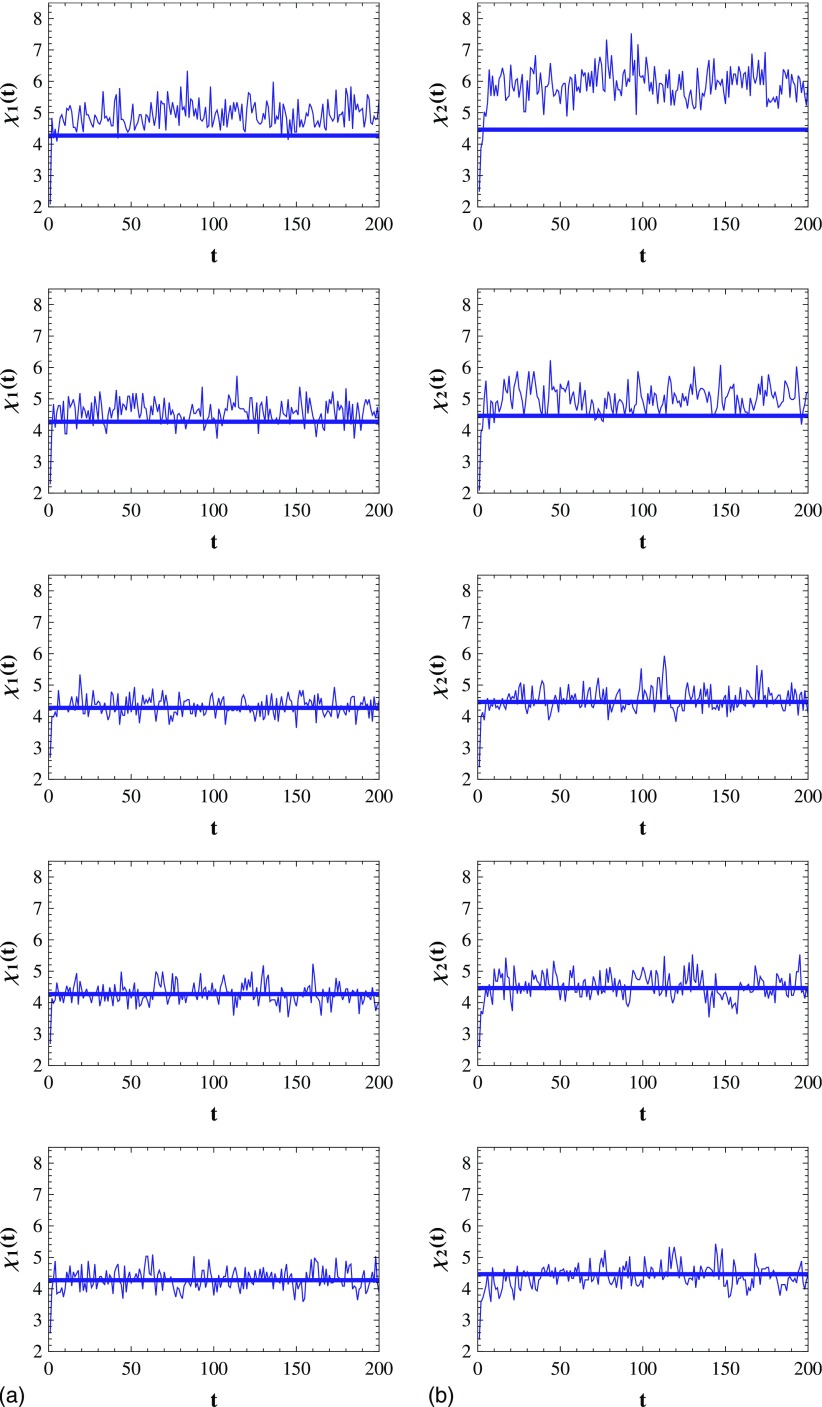
(a) The evolution of the CICS $\chi_{1}$ when $\rho_{1}=0.4$ in mode 1. v=0.2,1,2,6,10 (from top to bottom). The straight line corresponds to $\chi_{H}\left(t\right)$ with $\rho_{H}=0.4$ in homogeneous mode. (b) Same with (a) but for mode 2. Results are averaged over 15 runs.

In this section, we discuss the inhomogeneity of the epidemic spreading while the individuals walk randomly in the square (v>0), and let v∊[0.2,10]. As has been argued, L is set to 7, the initial proportions of the infected individuals in mode 1 and mode 2 are 10%. In Fig. 4, we plot the evolution of the infection density $\rho_{1}$ and $\rho_{2}$ in mode 1 and mode 2 in the case that v∊[0.2,10], respectively. As shown in Fig. 4, after the initial transient process, $\rho_{1}$ $\left(\rho_{2}\right)$ fluctuates narrowly around a value, which means that the SIS model reaches the steady state. Figure 5 shows the evolution of $\chi_{1}\left(t\right)$ and $\chi_{2}\left(t\right)$ in the case that v∊[0.2,10] and $\rho_{1}=\rho_{2}=0.4$.

**FIG. 6.**
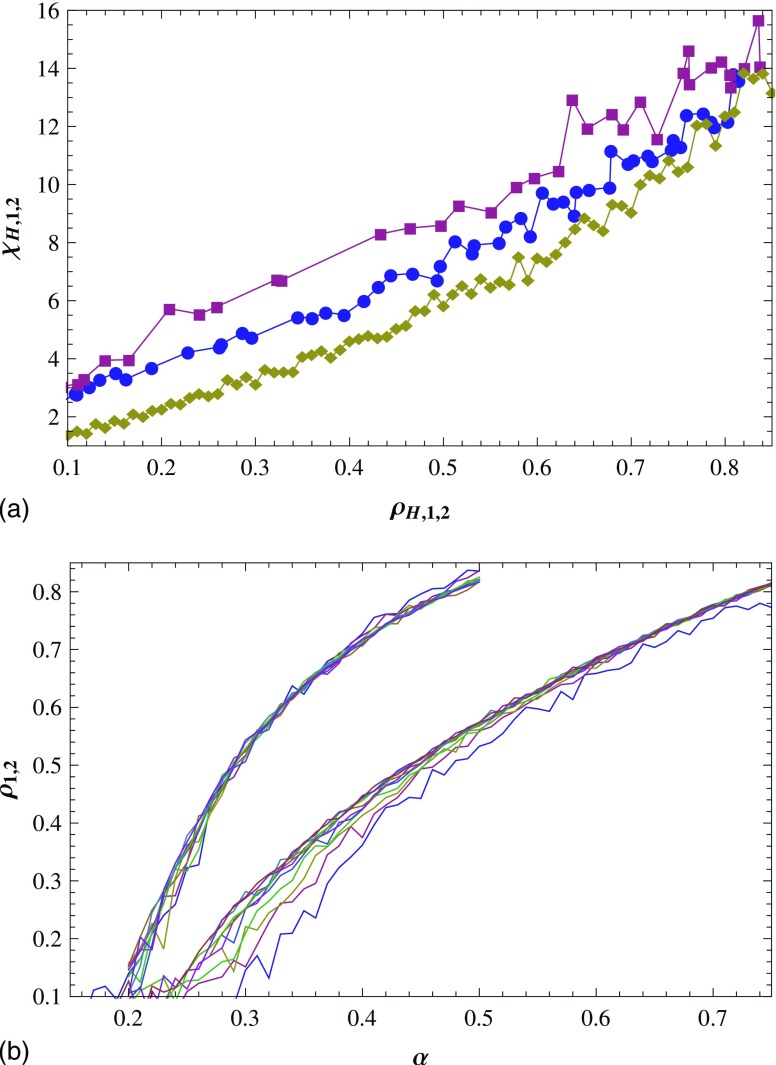
(a) When v=0.2, $\chi_{i}$ vs $\rho_{i}$, i=1,2 with the change of α: mode 1 (circles) and mode 2 (squares). Also, $\chi_{H}$ vs $\rho_{H}$ (diamonds). (b) $\rho_{i}$ vs α, i=1,2 for v∊[0,10]: mode 1 (the bottom curves) and mode 2 (the top curves). Results are averaged over 15 runs.

For each value of v, we can plot $\chi_{1}$ $\left(\chi_{2}\right)$ as a function of $\rho_{1}$ $\left(\rho_{2}\right)$ by changing the α value. Curves of $\chi_{i}$ versus $\rho_{i}$, i=1,2 at v=0.2 and the curve of $\chi_{H}$ versus $\rho_{H}$ [same with that in Fig. 3(a)] are given in Fig. 6(a). It shows that when $\rho_{1}$ $\left(\rho_{2}\right)∊\left[0.1,0.7\right]$, the epidemic spreading is inhomogeneous; when $\rho_{1}$ $\left(\rho_{2}\right)$ is about 0.8, the infected individuals are distributed homogeneously. Clearly, this conclusion is consistent with that of static networks, which are drawn in Fig. 3(a). The correspondences between $\rho_{1}$ $\left(\rho_{2}\right)$ and α for different v∊[0,10] are given in Fig. 6(b). Interestingly, the different v (including v=0) corresponds to the similar $\rho_{2}$ for a given α, as shown in Fig. 6(b). This means that the moving of individuals cannot affect the proportion of the infected individuals in mode 2. This conclusion can be obtained by letting the delay of the model τ=0 in Ref. 18.

**FIG. 7.**
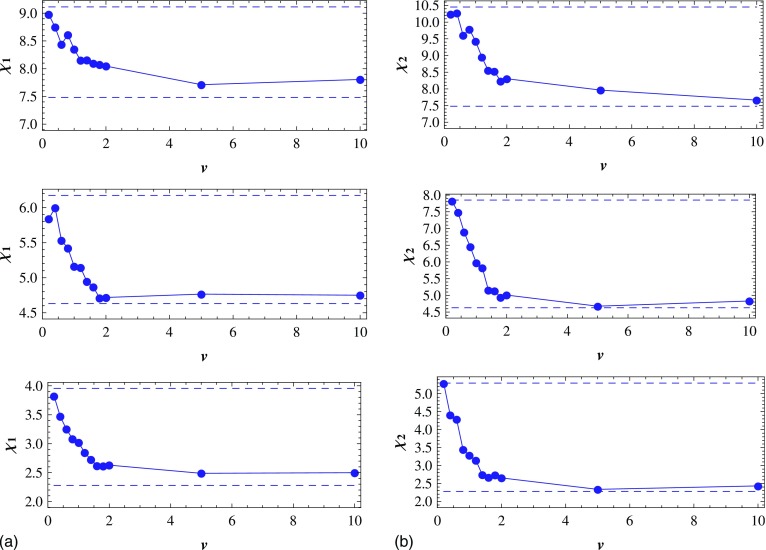
(a) In mode 1, the CICS $\chi_{1}$ as a function of speed v (v∊[0.2,10]) for the infection density $\rho_{1}=0.2,0.4,0.6$ (from bottom to top). Each bottom (top) dashed line corresponds to $\chi_{H}$ $\left(\chi_{1}\right)$ for the same $\rho_{1}$ in Fig. 3(a). (b) Same as (a) but for mode 2. Results are averaged over 15 runs.

In order to further study the impact of speed v on the inhomogeneity of the epidemic spreading, we will investigate the variation of $\chi_{1}$ $\left(\chi_{2}\right)$ with increasing of v for different $\rho_{1}$ $\left(\rho_{2}\right)$. As the epidemic spreading emerges the inhomogeneity when $\rho_{1}$ $\left(\rho_{2}\right)∊\left[0.1,0.7\right]$, $\rho_{1}$ $\left(\rho_{2}\right)$ are set to 0.2, 0.4, and 0.6 in the following discussion. At first, we plot $\chi_{1}$ as a function of $\rho_{1}$ by changing the value of α for each v. Then a set of functions of $\chi_{1}$ and $\rho_{1}$ is obtained by changing the v value. Corresponding to $\rho_{1}=0.2$, a set of $\left(v,\chi_{1}\right)$ can be gotten. Then $\chi_{1}$ is plotted as a function of v when $\rho_{1}=0.2$ in the bottom subplot of Fig. 7(a). For the sake of comparison, we plot two straight lines in the same subplot, which respectively correspond to $\chi_{H}$ (the bottom dashed line) and $\chi_{1}$ (the top one) for $\rho_{1}=0.2$ in Fig. 3(a). Similarly, the curves and straight lines corresponding to $\rho_{1}=0.4,0.6$ are also plotted in Fig. 7(a) (the middle subplot for $\rho_{1}=0.4$, the top subplot for $\rho_{1}=0.6$). In the same way, we plot $\chi_{2}$ as a function of v for $\rho_{2}=0.2,0.4,0.6$, respectively, and the corresponding straight lines in Fig. 7(b).

As can be seen in Fig. 7, when v∊[0.2,2], $\chi_{1}$ $\left(\chi_{2}\right)$ is apparently larger than $\chi_{H}$ for each $\rho_{1}$ $\left(\rho_{2}\right)$, which means that the epidemic spreading is inhomogeneous. Concretely, when v=0.2, $\chi_{1}$ $\left(\chi_{2}\right)$ is close to that of the static network (v=0). With increasing of v, $\chi_{1}$ $\left(\chi_{2}\right)$ decreases evidently and moves toward $\chi_{H}$ (homogeneous mode) progressively. This means that the inhomogeneity of the epidemic spreading becomes smaller with increasing speed v. Generally speaking, it is easier to maintain a structure in the static environment. Interestingly, our simulation results indicate that when the individuals walk randomly and the speed is not very high, their distribution is inhomogeneous. That is, the inhomogeneity of the epidemic spreading is kept in the dynamic environment. When v>2, $\chi_{1}$ $\left(\chi_{2}\right)$ is always near $\chi_{H}$, which means that the distribution of the infected individuals is approaching to that in homogeneous mode. This is because when v is large enough, any infected individual can easily jump out the area that is covered by its infected neighbors. Therefore, the large infected clusters cannot form.

As also shown in Fig. 7, the infection density affects the difference between the maximum and minimum of $\chi_{1}$ $\left(\chi_{2}\right)$. When $\rho_{1}=0.2$ $\left(\rho_{2}=0.2\right)$, the maximum of $\chi_{1}$ $\left(\chi_{2}\right)$ is 1.54 (2.26) times the minimum, while when $\rho_{1}=0.6$ $\left(\rho_{2}=0.6\right)$, the maximum of $\chi_{1}$ $\left(\chi_{2}\right)$ is 1.16 (1.34) times the minimum. Then we can say that the smaller the infection density $\rho_{1}$ $\left(\rho_{2}\right)$, the stronger v affects the inhomogeneity of the epidemic spreading. Besides, comparing Fig. 7(a) and Fig. 7(b) represents that when $\rho_{1}=\rho_{2}$ and v∊[0.2,2] are the same, $\chi_{2}$ are always larger than $\chi_{1}$. This means that the inhomogeneity is more obvious in mode 2 than in mode 1 at the same speed v, which is in accord with the case of static networks.

## V. CONCLUSIONS

In this paper, the inhomogeneity of the epidemic spreading in two spreading modes of the SIS model is investigated. The simulations in the static and dynamic networks show that the infected individuals are usually prone to gather into large clusters as the infected individuals always infect their neighbors. For such a reason, the epidemic usually spreads inhomogeneously. Even in dynamic networks, the inhomogeneity can be kept well. And, the smaller the infection density, the more inhomogeneously the epidemic spreads. However, the inhomogeneity decreases with the increase of the individuals’ speed in the dynamic networks, and the epidemic spreading becomes almost homogeneous when the speed is large enough.
